# Supplementary figures and images for: Integrated microRNA and proteome analysis of cancer datasets with MoPC
Source: PLoS One. 2024 Mar 21;19(3):e0289699. doi: 10.1371/journal.pone.0289699 (PMC10956802; doi:10.1371/journal.pone.0289699)

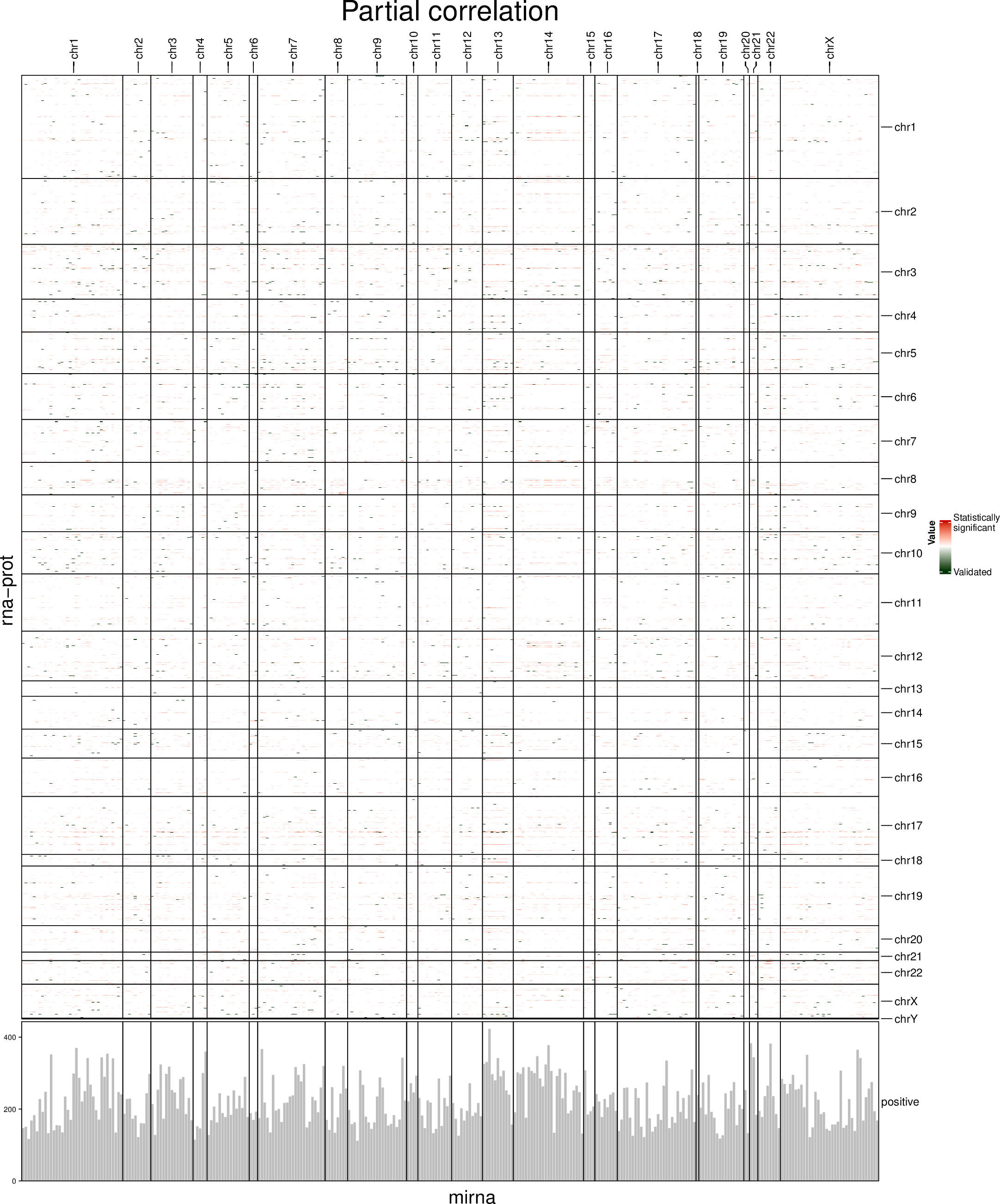

Supplement: S1 Fig — The heatmap reports in red the miRNA-genes pairs resulting significantly with the MoPC analysis. In dark green, the miRNA-genes pairs are statistically significant in the input dataset and validated in at least one of the three databases (miRDB, TargetScan, and miRTarBase). Genes are reported on the rows, and miRNAs on the columns. Both genes and miRNAs are chromosomally ordered. Only the 5p’ most expressed miRNAs in breast cancer, according to isomiRTar [6], have been reported in the columns to provide more understandable results. (TIF) [file pone.0289699.s001.tif]

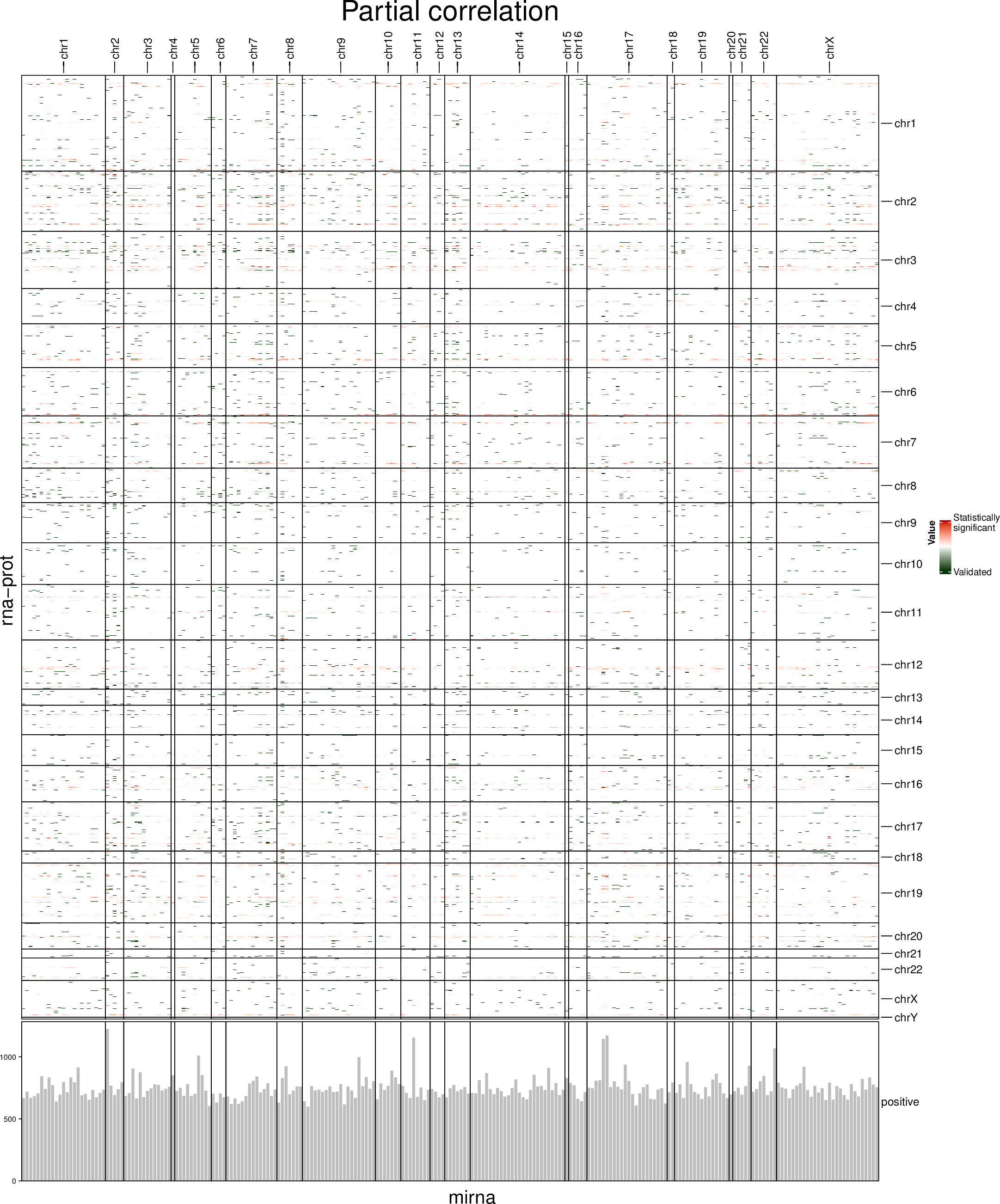

Supplement: S2 Fig — The heatmap reports in red the miRNA-genes pairs resulting significantly with the MoPC analysis. In dark green, the miRNA-genes pairs are statistically significant in the input dataset and validated in at least one of the three databases (miRDB, TargetScan, and miRTarBase). Genes are reported on the rows, and miRNAs on the columns. Both genes and miRNAs are chromosomally ordered. Only the 25% most expressed miRNAs in this glioblastoma dataset have been reported in the columns to provide more understandable results. (TIF) [file pone.0289699.s002.tif]

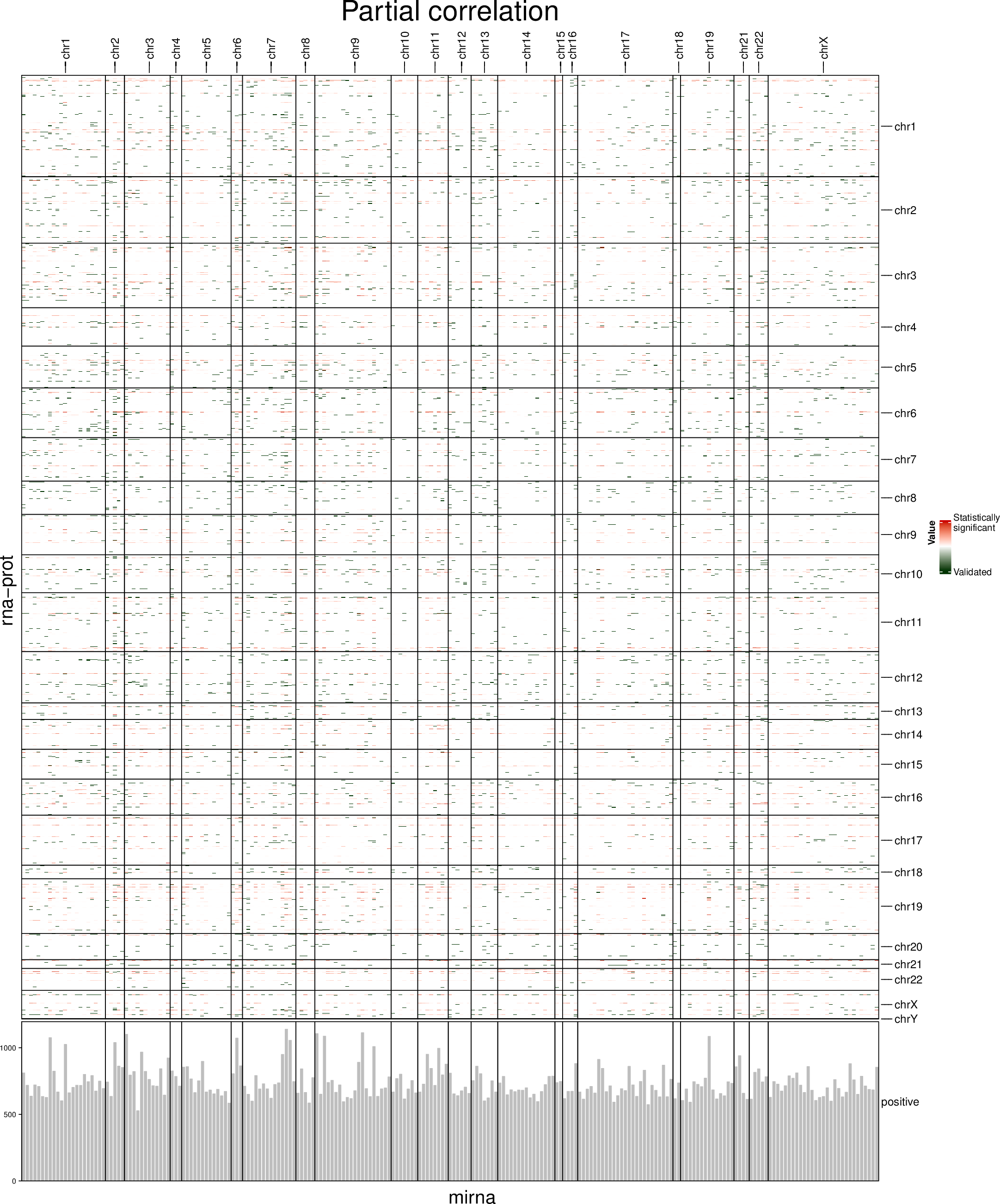

Supplement: S3 Fig — The heatmap reports in red the miRNA-genes pairs resulting significantly with the MoPC analysis. In dark green, the miRNA-genes pairs are statistically significant in the input dataset and validated in at least one of the three databases (miRDB, TargetScan, and miRTarBase). Genes are reported on the rows, and miRNAs on the columns. Both genes and miRNAs are chromosomally ordered. Only the 5p’ most expressed miRNAs in lung cancer, according to isomiRTar [6], have been reported in the columns to provide more understandable results. (TIF) [file pone.0289699.s003.tif]
